# Gut Microbial-Derived Metabolites as Immune Modulators of T Helper 17 and Regulatory T Cells

**DOI:** 10.3390/ijms24021806

**Published:** 2023-01-16

**Authors:** Laura Calvo-Barreiro, Longfei Zhang, Somaya A. Abdel-Rahman, Shivani Paritosh Naik, Moustafa Gabr

**Affiliations:** 1 Department of Radiology, Molecular Imaging Innovations Institute (MI3), Weill Cornell Medicine, New York, NY 10065, USA; 2 Department of Medicinal Chemistry, Faculty of Pharmacy, Mansoura University, Mansoura 35516, Egypt

**Keywords:** short-chain fatty acids, indole, polyamines, choline, secondary bile acids, Treg cells, Th17 cells, immune regulation

## Abstract

The gut microbiota and its derived metabolites greatly impact the host immune system, both innate and adaptive responses. Gut dysbiosis and altered levels of microbiota-derived metabolites have been described in several immune-related and immune-mediated diseases such as intestinal bowel disease, multiple sclerosis, or colorectal cancer. Gut microbial-derived metabolites are synthesized from dietary compounds ingested by the host or host-produced metabolites, and additionally, some bacterial products can be synthesized de novo. In this review, we focus on the two first metabolites families including short-chain fatty acids, indole metabolites, polyamines, choline-derived compounds, and secondary bile acids. They all have been described as immunoregulatory molecules that specifically affect the adaptive immune system and T helper 17 and regulatory T cells. We discuss the mechanisms of action and the consequences in health and diseases related to these gut microbial-derived metabolites. Finally, we propose that the exogenous administration of these molecules or other compounds that bind to their immunoregulatory receptors in a homologous manner could be considered therapeutic approaches.

## 1. Introduction

Living organisms sustain life by having a defense mechanism against microbial assaults. Different life forms have developed and evolved strategies that limit the invasion of microorganisms into the host. Specifically, mammals have an immune system comprising innate and adaptive immunity [1]. The innate immune system acts as the first line of defense through invariant pattern recognition receptors (PPRs) that are equipped to identify pathogen-associated molecular patterns (PAMPs) [1,2]. In contrast, the adaptive immune system comprising T and B cells, among others, form the highly specific second line of defense. In particular, T cells originate from hematopoietic stem cells present in the bone marrow, followed by their migration to and maturation in the thymus [1].

The gastrointestinal tract is the largest surface in humans to come into straight contact with the outside environment [3]. This leads to the gut mucosa being exposed to a large variety of external antigens. T helper (Th) cells come into play when antigen-presenting cells (APCs) identify microorganism antigens, leading to cytokine production and, eventually, the activation and differentiation of Th cells [3]. In contrast, regulatory T (Treg) cells counterbalance the effector responses of Th cells to minimize any collateral damage after Th activation [3]. In the gut, the harmonic balance of Th/Treg cells is usually attained by in situ induction of these cells from naïve T cells, recruitment of differentiated Th/Treg cells into the tissue, and reprogramming of already differentiated Th/Treg cells towards other lineages in peripheral tissues [4].

CD4^+^ T cells are split into two subclasses: Th and Treg cells. Th cells are an essential part of the adaptive immune system in synchronizing the defense against pathogens. They play an important role through their distinctive cytokines and effector functions in guiding tissue inflammation. Within the Th subsets, Th1 cells produce interferon-gamma (IFN-γ) as protection against intracellular pathogens, Th2 cells produce interleukin 4 (IL-4), IL-5, and IL-13 to clear parasitic pathogens, and Th17 cells produce IL-17, IL-21, and IL-22 to control microbial pathogens [5]. Moreover, Th17 cells have been researched extensively to uncover their role in health and disease and are associated with tissue inflammation and autoimmune disorders such as multiple sclerosis (MS) [6,7,8], inflammatory bowel disease (IBD) [9,10,11], or rheumatoid arthritis [12,13], among others. In contrast, Treg cells are responsible for repressing any probable detrimental Th cell activities [14]. The concept of suppressor T cells, being able to downregulate the activity of other T cells, was already around in the 1970s. However, it was only around the mid-1990s that the concept of Treg cells as a new subpopulation of CD4^+^ T cells was acknowledged [15]. This development led to a clear division of lineages of CD4^+^ T cells into T helper (Th1, Th2, and Th17) and Treg cells. However, much is still unknown about Treg cell biology, which attracts controversy and debate. 

Treg cells are produced in the thymus (natural Tregs, nTregs) or the periphery (induced Tregs, iTregs) and are recognized as the primary holders of peripheral tolerance and staunch suppressors of inflammation [16]. Although nTregs and iTregs have commonality in function, there are differences in phenotype as well as considerable ones in inherent stability and transcriptional and epigenetic status. The gut is one such organ in the body exposed to many foreign antigens and is therefore influenced by Tregs produced because of peripheral and not thymic differentiation. Both Th17 and Tregs have shown commonality despite their functional dissimilarities and are highly represented in the periphery of the intestine [17]. They comprise miscellaneous subpopulations with the ability to alter suppressor or effector capabilities in different circumstances [17]. In addition, both share notable mediators and mechanisms that regulate them [18].

The compelling evidence obtained over the last few decades clearly points towards Treg and Th17 cells being involved in maintaining immune homeostasis. Indeed, a disrupted Th17/Treg balance is linked to autoimmunity, cancer, and metabolic diseases [18,19,20]. Research also strongly points towards the connection of the gut microbial community being essential to the sustenance of a healthy host [3]. Clear links have been shown between the gut microbiota and the central nervous system (CNS), the cardiovascular system, or intestinal inflammation [21,22,23]. Thus, alterations in microbial composition, also known as gut dysbiosis, are related to several chronic diseases [24]. Diseases arising from this are defined by immune alterations, such as major disturbances in the Th/Treg balance, and are not limited to the GI tract but also affect central/peripheral tissues and can reach systemic circulation.

Finally, it is still unknown if the remodeling of the T cell landscape is controlled by the gut microbiota and involves epigenetic mechanisms. Therefore, could novel strategies such as gut microbiota manipulation redesign T-cell epigenome to contain inflammatory diseases [4]? The evidence obtained so far can explain the mechanisms behind Th/Treg balance and how they can be a potential target in inflammatory disease treatment. However, there has been significant research pointing towards the close association of gut microbiota with Th17/Treg balance and how gut microbiota-derived metabolites, such as short-chain fatty acids (SCFAs) or bile acids, among others, are involved in the differentiation of Th17 and Treg cells.

## 2. Gut Microbiota-Derived Metabolites: Effect on Th17/Treg Balance and Disease

### 2.1. Produced by Bacteria from Dietary Components

#### 2.1.1. Short-Chain Fatty Acids

SCFAs are gut microbiota-derived products mainly produced from the fermentation of digestion-resistant oligosaccharides and dietary fiber. SCFAs are saturated hydrocarbons composed of one to five carbons bound to a carboxyl group, with acetate (C2), propionate (C3), and butyrate (C4) being the most commonly studied ones.

Seminal work performed by Garrett and collaborators showed how SCFA supplementation (acetate, propionate, butyrate, or a combination of all) in the diet of germ-free (GF) mice increased Treg cells in the colon (cTreg) [25]. SCFAs could also increase the number and percentage of IL-10-producing cTreg cells in specific pathogen-free (SPF) mice but no other inflammatory populations such as Th1 or Th17 [25]. They further demonstrated that these immunoregulatory cells were not generated de novo but expanded from a nTreg cells present in the colonic lamina propria [25]. By the time SCFAs were being studied as immunoregulatory molecules, prior studies had already described some of their receptors: G protein-coupled receptor (GPR) 41, GPR43, or GPR109A [26,27,28,29], as well as intracellular activated pathways: production of inositol 1,4,5-trisphosphate, intracellular Ca^2+^ release, and phosphorylation of p42 and p44 mitogen-activated protein kinases (ERK1/2), inhibition of cAMP accumulation, or inhibition of histone deacetylase (HDAC) [27,28,30]. GPR41 is mainly present in the adipose tissue, pancreas, spleen, and peripheral blood mononuclear cells (PBMCs), and GPR43 is present in neutrophils, monocytes, PBMCs, and the spleen [26,27]. In contrast to the broad expression pattern of GPR41, the specialization of GPR43 in immune populations suggests a role in the recruitment of these cells toward bacterial niches. Indeed, this pioneer investigation described how the effect of propionate on cTreg cells depended on HDAC inhibition via the Ffar2 gene, which encodes GPR43 [25].

Since SCFAs are produced by gut microbiota, it was expected that further investigation on GF animals or mice treated with broad-spectrum antibiotics highlighted a significant reduction in propionate and butyrate in fecal extracts [31]. Propionate and butyrate supplementation, but not acetate, for naïve CD4^+^ T cells increased the number of FoxP3 cells in vitro in a TGFβ-dependent way [31,32]. Interestingly, Haghikia and collaborators also showed that naïve CD4^+^ T cells treated with propionate under polarizing conditions promoted Treg cell populations and suppressed the Th17 response, but medium- and long-chain fatty acids (MCFAs and LCFAs, respectively) promoted their differentiation to Th1 and Th17 cells [33].

In vivo experiments in mice treated with broad-spectrum antibiotics corroborated that propionate and butyrate administration stimulated extrathymic, but not thymic, generation of Treg cells, while acetate and propionate could increase cTreg cells [31]. In contrast, as previously shown, another coetaneous article proved how butyrate did increase cTreg cells when SPF mice were orally treated with this SCFA [32]. However, treatment of these SPF animals with butyrate did not affect the accumulation of Th1, Th2, or Th17 cells in the colonic lamina propria [32]. Again, if GF animals, instead of SPF or monocolonized GF mice, were treated with SCFA butyrate, no increase in cTreg cells was observed [32]. This latter result highlights how commensal bacteria specifically, and not just their subproducts or metabolites, might be essential for Treg cell induction. Then again, in vitro treatment of naïve CD4^+^ T cells showed how butyrate enhances histone acetylation in the regulatory regions of the Foxp3 locus, which in turn epigenetically upregulates *FOXP3* gene, but how TLR-MyD88 signaling, which has been involved in Treg cell expansion [34], is dispensable for butyrate-dependent Treg cell differentiation [32]. Recently, all these results were partially proven on human cells, and propionate and butyrate, but not acetate, increased in vitro differentiation to Treg cells from human naïve CD4^+^ T cells and enhanced their immunosuppressive capacity when co-cultured with allogeneic CD4^+^CD25^−^ responder cells [35].

In vivo, dendritic cells (DCs) and intestinal epithelial cells (IECs) are the two primary sources of TGFβ1 in the gut [36,37], which make these two cell populations important for the regulation of Treg cell production. Interestingly, both propionate and butyrate were also capable of enhancing DC capability to increase the FoxP3^+^ population in vitro [31]. Again, results pointed out to the SCFA capability of inhibiting HDAC activity which, in turn, would make DCs increase Treg cell differentiation [31]. Latter research on butyrate also showed its effect in DCs and colonic macrophages through GPR109A binding and signaling in the colon and how these two cell types were important for promoting IL-10-producing T cells and suppressing colitis and experimental colon cancer [38]. Finally, regarding the SCFA effect on IECs, butyrate has been described to be GPR41, GPR43, and GPR109A independent but to promote TGFβ1 expression through HDAC inhibition [39].

Finally, it has been reported that SCFA and gut microbiota populations, which are great producers of these fatty acids, such as *Clostridium*, *Faecalibacterium*, or *Roseburia*, among others, [40] present lower levels in patients with IBD [41,42,43] as well as in patients with MS [44,45,46,47] or colorectal cancer [48,49,50] compared to healthy individuals. Their beneficial effect has also been demonstrated in experimental models such as colitis [25,32,51], experimental autoimmune encephalomyelitis (EAE) [33,52], allergic asthma [53], arthritis [51,54], and prostatitis [55], among others. Thus, this positive effect that has been partly attributed to the induction of Treg cells could be translated into the clinic, and in fact, a few clinical trials have shown some preliminary but encouraging results [56,57,58].

#### 2.1.2. Indole Metabolites

**Figure 1:**
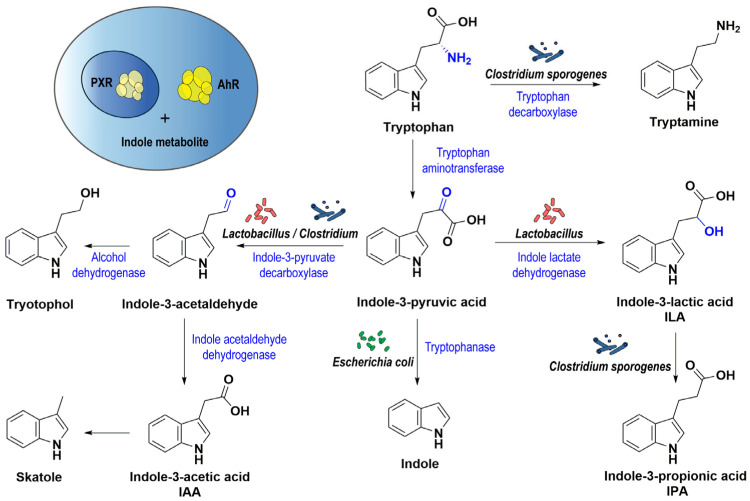
Production of indole metabolites by gut microbiota and its corresponding enzymes. Indole metabolites bind to intracellular or cytoplasmic transcription factor PXR or AhR, respectively, and activate their corresponding pathway. Tryptophan is transformed into different indole metabolites via microbial enzymes (blue color) present in specific gut microorganisms. Abbreviations: AhR: aryl hydrocarbon receptor; PXR: pregnane X receptor.

Colonic indoles are gut bacterial metabolites derived from tryptophan [59]. Excess unabsorbed tryptophan passes to the large intestine, and it is biotransformed into different bioactive indole derivatives through the action of tryptophanase enzyme and other bacterial catalytic enzymes, which are found in many gut bacterial species, such as: *Escherichia coli*, *Micrococcus aerogenes*, *Paracolobactrum coliforme*, *Proteus vulgaris*, and different species of genera *Clostridium* and *Lactobacillus* [60,61,62,63,64,65]. Indole-3-acetic acid (IAA), indole-3-propionic acid (IPA), indole-3-lactic acid (ILA), indoxyl sulfate, skatole, and tryptamine are examples of tryptophan-derived indoles that might enter the host circulation and influence different body systems and organs [66,67,68,69]. Individuals with different gut bacteria may have different indole metabolites, which are associated with the pathogenesis of cardiovascular, metabolic, and other diseases (Figure 1) [70].

The bioactive indole metabolites might regulate intestinal immune response or mucosal integrity through binding to aryl hydrocarbon receptor (AhR) or pregnane X receptor (PXR) (Figure 1) [71,72]. Indole metabolites elicit nuclear translocation of AhR, which correlates with their roles in intestinal health and disease [71]. In addition, the activation of AhR leads to increased colonic *IL-22* mRNA expression, which plays an important role in intestinal homeostasis (Figure 1) [73].

Indole bacterial metabolites are involved in the differentiation of Th17 and Treg cells by augmenting TGF-β-induced Treg expansion, function, and stability, thus affecting many immune-related diseases [20,74,75]. Shen and collaborators investigated the relationship between IAA and ankylosing spondylitis (AS) in mice. The study revealed that IAA decreases the severity of AS by enhancing the production of IL-10 while reducing the production of tumor necrosis factor-alpha (TNFα) [76]. Moreover, IAA activates the AhR pathway, increases Treg cells, and inhibits Th17 cells [77].

Another important indole metabolite is IPA, which displays antimycobacterial and antiinflammatory activities [78]. IPA exerts its activity via the activation of AhR, leading to further regulation of many immune genes involved in inflammation [79]. Low fecal levels of IAA and low serum levels of IPA were found in patients with IBD, accompanied by high serum levels of tryptophan, which might indicate dysregulation of this amino acid in patients [80]. Lower blood levels of IPA were also reported in patients with liver fibrosis, Huntington’s disease, and active colitis compared to healthy individuals [81,82,83]. Moreover, it was proven to enhance vascular health, while indoxyl sulfate promoted vascular inflammation [84].

The gut bacterial ILA was proven to affect the transformation of naïve CD4^+^ T cells into Treg cells and suppress Th17 cell differentiation in vivo, which plays an essential role in autoimmune and inflammatory diseases [85]. Moreover, gut bacterial indoles are involved in many cancer types, such as colon carcinogenesis and metastasis of renal cell carcinoma, which is promoted by the activation of AhR by bacterial indoles [86,87].

#### 2.1.3. Polyamines

Polyamines are a class of low molecular weight aliphatic compounds with multiple amino groups, which are widely distributed in eukaryotic cells within millimolar concentrations [88]. In mammalian cells, the naturally occurring polyamines consist of spermine, spermidine, and putrescine. Since the amino group tends to be protonated under the physical pH level, polyamines are generally positively charged in animals, which facilitates their interaction with anionic biomacromolecules (such as, DNA, RNA, and proteins), thus participating in various biological processes: cell proliferation and differentiation, and apoptosis [88,89,90]. Moreover, polyamines are also involved in ion-channel regulation, gene transcription and translation, and the maintenance of biological membrane and chromosome stability [90,91]. Although intracellular polyamines homeostasis is strictly regulated, the alteration in polyamines content and metabolism can be observed in both normal biological and pathological processes, such as T cell activation and carcinogenesis, respectively.

Even though polyamines could be supplied by de novo synthesis in cells, diet and gut microbiota are still considered the primary source of polyamines in the human body [92]. Polyamines can be found in almost all food, but they are particularly abundant in fresh meat, cheese, and soybeans. Besides dietary sources, genera *Bifidobacterium*, *Bacteroides*, *Clostridium*, *Enterococcus*, *Lactobacillus*, *Enterobacter*, *Streptococcus*, and *Escherichia* are the main microorganisms that synthesize polyamines for sustaining their growth and host immune modulation [93,94]. As a result of food digestion and microbiota production, polyamines are present in the intestinal tract at a millimolar concentration, which could be absorbed by IECs and later transported to the circulation system via the portal vein [88].

While T cell activation requires the upregulation of polyamines levels, different Th subsets have varying degrees of dependency to extracellular polyamines supplements in in vivo experiments [95]. Puleston and colleagues treated four subsets of Th cells (Th1, Th2, Th17, and Treg) with different radionuclide-labeled precursors of polyamines biosynthesis, and found that Th17 and Treg cells presented lower radioactive activity than others, indicating the diminished biosynthesis of polyamines in Th17 and Treg cells, which are more sensitive to the disturbance of polyamines concentration in the surrounding microenvironment [95]. In vitro incubation of murine CD4^+^CD25^−^ T cells with three different polyamines under Th17 cell-polarizing conditions showed a significant increase in the frequency of FoxP3^+^ cells and a reduction in IL-17 production in both spermidine and spermine groups while no difference was observed in the putrescine group [93]. Moreover, cell viability was not affected by the presence of these three polyamines, suggesting that a change in the Th17/Treg ratio was accomplished via a phenotype shift effect, instead of modifying the viability of Th17 cells. Besides promoting Treg cells in in vitro experiments, spermidine also displayed the ability to alleviate disease severity in animal models by skewing the Th17/Treg ratio [93]. In an experimental murine model of colitis, 21-day oral treatment with spermidine alleviated signs of colitis such as body weight loss, colon shortness, and diffuse cell infiltrates in the colon. Moreover, an increase in both the frequency and number of FoxP3^+^ cells together with a slight reduction in IL-17 production was observed in the colon lamina propria of mice treated with spermidine supplement [93]. Additionally, oral administration of spermidine to EAE mice was reported to alleviate disease signs by decreasing IL-17 production [96].

Even though spermidine could create an antiinflammatory environment by altering the Th17/Treg ratio, the mechanism behind this phenomenon remains elusive and several hypotheses were developed to address this question. Yang and collaborators transferred macrophages derived from spermidine-treated EAE mice into new EAE animals and observed alleviated signs in recipient individuals [96]. Therefore, they speculated that macrophages could be the linker between spermidine and CD4^+^ T cell differentiation [96]. After isolating macrophages from spermidine-treated EAE mice, they found that the activity of NF-κB (a critical regulator of proinflammatory responses) was dramatically reduced and accompanied by a decrease in the phosphorylation of p65, which is responsible for the post-translational modifications of NF-κB [96]. Additionally, the upregulation of arginase-1 activity was also detected in macrophages, which is a highly expressed enzyme in the M2 antiinflammatory phenotype [96], and the inhibition of this enzyme halted the therapeutic effect of spermidine [96]. It was finally inferred that spermidine could induce arginase-1 and inhibit the NF-κB signaling pathway by restricting the activity of p65. These, in turn, could drive macrophages towards M2 polarization, thus affecting T cell differentiation toward antiinflammatory responses [96]. Recently, the mechanism of Th17 promotion in the murine intestinal tract by *Eggerthella lenta* was discovered. It was found that Cgr2, a gut bacterial enzyme, could increase the expression of RAR-related orphan receptor gamma t (Rorγt) by metabolizing some unknown inhibitors, thus leading to inflammation in the colon [97]. They also revealed that oral administration of L-arginine (a precursor of polyamines biosynthesis) to *Eggerthella lenta*-colonized mice could alleviate colitis severity by blocking Cgr2, which was speculated to be due to polyamines suppressing Th17 differentiation [97]. Furthermore, Carriche and collaborators found that spermidine could inhibit mTOR, which might influence T cell differentiation [93].

In addition to extracellular polyamines, intracellular polyamines biosynthesis in T cells is also upregulated after activation to sustain their proliferation, differentiation, and functional secretion of cytokines, while the dysfunction might lead to severe transcriptional perturbations [98,99]. Puleston and coworkers revealed aberrant functions across all four subsets of Th cells after deleting the enzyme ornithine decarboxylase (ODC), a rate-limiting enzyme in polyamines biosynthesis [95]. One of these altered functions included reduced IL-17 production and Rorγt expression and increased FoxP3 expression in Odc^−/−^ Th17 cells [95]. However, no change in FoxP3 or Rorγt expression in Odc^−/−^ Treg cells was observed [95]. After inhibiting ODC activity by the oral administration of DFMO (an irreversible inhibitor of ODC) in EAE mice, Wagner and collaborators detected a significant increase in the frequency of FoxP3^+^ CD4^+^ T cells in the CNS of treated mice, indicating that ODC inhibition was also capable of promoting Treg cell differentiation in vivo [100]. However, like spermidine and spermine, the regulatory mechanism of ODC inhibition on CD4^+^ T cell differentiation is also unclear. Using the ATAC-seq assay, it was detected that the accessibility to Th17- and Treg-specific regions in the chromosome of both subsets were restricted and elevated, respectively, suggesting that abnormal CD4^+^ T cell differentiation is based on epigenetic remodeling [100]. Interestingly, Puleston and collaborators detected an alteration in cellular epigenetic status after ODC deletion and identified H3k9Ac and H3k27Ac as the dysregulated epigenetic marks [95]. After reducing the acetylation of H3k9 or H3k27 by chemical or genetic methods, the aberrant production of IFNγ in Odc^−/−^ Th17 cells was successfully reduced but failed in restoring the expression of IL-17 or Rorγt [95]. Moreover, they also found that spermidine deficiency was responsible for the shortage of eIF5A (a translation elongation factor) hypusination in Odc^−/−^ CD4^+^ T cells [95]. Based on the above data, a possible pathway of the polyamines-hypusine axis was proposed in controlling CD4^+^ T cell differentiation: as a result of the genetic deletion of ODC, the hypusine biosynthesis was restricted, which reduced eIF5A hypusination, thus leading to a series of alternations in epigenetic status, and finally resulting in the dysregulated expression of cytokines in Odc^−/−^ CD4^+^ T cells [95].

Polyamines derived from gut microbiota metabolism are an important source of exogenous supplements in humans, which are involved in the crosstalk between the microbiota and the host and participate in various biological processes. The role of polyamines in immune modulation has attracted intense research interest in recent years [88,91]. However, owing to the complicated regulatory network between polyamines and various enzyme activities, even though both spermidine and spermine could create an antiinflammatory environment by leaning Th17/Treg balance towards the Treg cell population, the molecular mechanism remains elusive [93,96]. Interestingly, even though only a few studies have shown the effect of putrescine in CD4^+^ T cell differentiation, the aberrant cytokine production caused by ODC deletion could be restored by simply adding putrescine to culture media, indicating that putrescine deficiency might be the direct cause of the epigenetic status changes in the Odc^−/−^ CD4^+^ T cells.

#### 2.1.4. Microbial Choline Metabolism

Anaerobic metabolism of choline, an essential nutrient found naturally in foods, by gut bacteria has been associated with various diseases, such as atherosclerosis, chronic kidney disease (CKD), and type 2 diabetes [101,102]. Patients with CKD have elevated circulating levels of trimethylamine N-oxide (TMAO) [101,102,103], a metabolite derived from the metabolism of choline, produced by the gut microbiome, and associated with cardiovascular diseases [104,105,106,107]. High circulating levels of TMAO predict increased risk for the development of CKD in healthy subjects [108], and are associated with decreased kidney function in mice [109]. Furthermore, TMAO-induced alloreactive T cell proliferation and differentiation into proinflammatory T helper subtypes, Th1 and Th17, were associated with increased severity of graft-versus-host disease (GVHD) [110].

**Figure 2:**
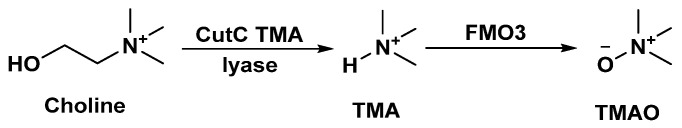
Two-step metabolic pathway for choline by CutC TMA lyase and FMO3 enzymes. Abbreviations: CutC: trimethylamine-lyase; FMO3: monooxygenase 3; TMA: trimethylamine; TMAO: trimethylamine N-oxide.

Anaerobic choline metabolism mediated by gut microbial trimethylamine (TMA)-lyase (CutC) is considered the major source of TMAO in the human body [111,112,113]. Thus, CutC TMA lyase has emerged as a promising therapeutic target for cardiovascular diseases and CKD [114,115]. The metabolic pathway of choline by gut bacteria proceeds through the cleavage of the C-N bond in choline via CutC TMA lyase to generate TMA and acetaldehyde (Figure 2) [112]. The produced TMA is further oxidized by hepatic flavin-dependent monooxygenase 3 (FMO3) to produce TMAO (Figure 2) [116]. Targeted inhibition of TMA lyase in a murine model of CKD resulted in the reduction in renal tubulointerstitial fibrosis and functional impairment [117]. In addition, TMA lyase inhibitors have demonstrated the ability to prevent thrombosis formation in mice fed with choline-supplemented diet [118].

**Figure 3:**
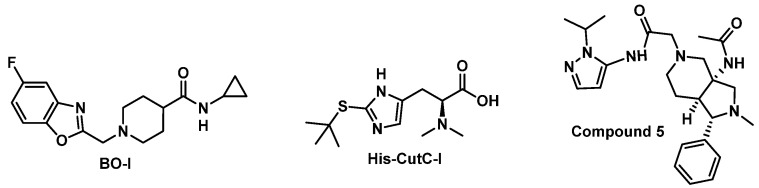
Chemical structures of BO-I, His-CutC-I, and compound 5.

Hazen and coworkers reported 3,3-dimethyl-1-butanol (DMB) as a first-in-class inhibitor of microbial choline TMA lyase activity [119]. However, the limited therapeutic potential of DMB has been demonstrated by the inability of a high dose of DMB (1.3% vol/vol) to fully rescue in vivo thrombosis in mice fed with a high-choline diet compared to chow-fed animals [118]. Efficient in vivo inhibition of TMA lyase by small molecules is currently restricted to choline analogs that function as covalent inhibitors of TMA lyase (e.g., iodomethylcholine (IMC)) [118,120,121]. We have initiated a series of focused screening campaigns of chemical libraries to identify new scaffolds as non-covalent inhibitors of TMA lyase [122,123,124]. As shown in Figure 3, our studies led to the discovery of several hit compounds (BO-I, His-CutC-I, and Compound 5) with intestinal metabolic stability and TMA lyase inhibition in human fecal samples [122,123,124]. Our workflow starts with subjecting a focused library to in vivo metabolic stability screening using the mixed gender human intestinal S9 fraction, followed by screening metabolically stable compounds for the ability to reduce the CutC-mediated production of TMA. Evaluation of the CutC inhibitory activity of BO-I in a dose-dependent assay demonstrated a half maximal inhibitory concentration (IC50) value of 2.4 ± 0.3 µM [122]. Kinetic analysis revealed that BO-I functions as a non-competitive inhibitor of CutC based on the unchanged Michaelis constant (K_M_) as well as the reduced V_max_ (maximum rate for enzymatic reaction) from the Lineweaver–Burk plot (the double reciprocal plot of reaction velocity versus the substrate concentration). Interestingly, BO-I blocked the transformation of choline to TMA in whole-cell assays of multiple bacterial strains [122]. Therefore, these hits represent promising starting points for hit-to-lead optimization, and further evaluation is needed regarding their ability to reduce renal tubulointerstitial fibrosis and thrombosis in animal models.

### 2.2. Produced by the Host and Modified by Gut Microbiota

#### Secondary Bile Acids

**Figure 4:**
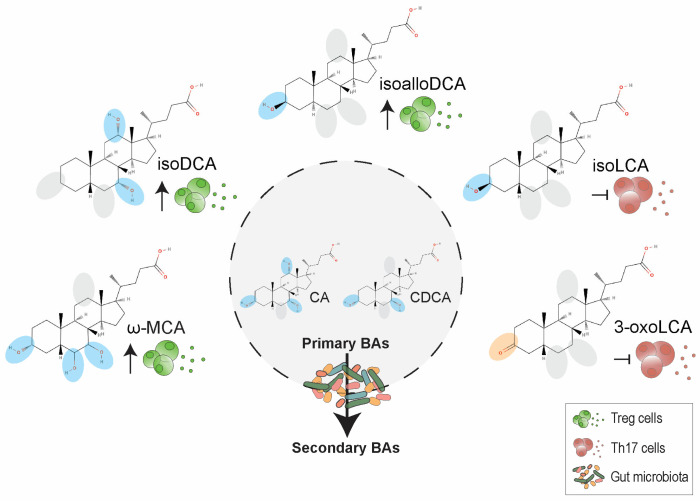
Secondary bile acids have immunoregulatory and antiinflammatory effects on T cell populations. Primary bile acids (BAs), such as cholic acid (CA) and chenodeoxycholic acid (CDCA), are transformed into secondary BAs by the gut microbiota. Only a few secondary BAs have been proven to exert immunoregulatory (increase of regulatory T cell populations) or antiinflammatory (inhibition of proinflammatory T helper 17 cells) effects among the overall set of secondary BAs (more than 20). For visualization purposes, hydroxyl radicals are highlighted in blue color, carbonyl group in orange color, and the absence of radical in grey color. Abbreviations: BA: bile acid; CA: cholic acid; CDCA: chenodeoxycholic acid; DCA: 3α,12-dihydroxy-5β-cholan-24-oic acid; LCA: lithocholic acid; MCA: muricholic acid.

Primary bile acids (BAs) are cholesterol metabolites that are synthesized de novo by hepatocytes [125]. The two primary BAs in human liver are cholic acid (CA; 3α,7α,12α-trihydroxy-5β-cholan-24-oic acid) and chenodeoxycholic acid (CDCA; 3α,7α-dihydroxy-5β-cholan-24-oic acid) (Figure 4). Prior to their secretion into bile, primary BAs are conjugated to glycine or taurine to decrease toxicity and increase solubility and transformed into bile salts (Na^+^ or K^+^ ions) [125]. Once bile enters the small intestine at the duodenum, BAs help to absorb and transport nutrients, lipids from the diet, and fat-soluble substances such as vitamins [126].

Once these primary BAs reach the distal ileum and colon, the commensal or gut microbiota take action. On the one hand, the commensal microbiota deconjugates glyco- and tauro-conjugated CA and CDCA, and on the other hand, further removes the 7α-hydroxy group from deconjugated primary BA to form secondary BAs deoxycholic acid (DCA; 3α,12-dihydroxy-5β-cholan-24-oic acid) and lithocholic acid (LCA; 3α-monohydroxy-cholanoic acid), respectively, or perform a 7β-hydroxyl epimerization of CDCA to form secondary BA ursodeoxycholic acid (UDCA; 3α,7β-dihydroxy-5β-cholan-24-oic acid) (Figure 4) [127]. Further deconjugated primary BAs also undergo different microbial biotransformations in the colon, such as dehydrogenation, dehydroxylation, and epimerization, leading to more than 20 different secondary BAs [127]. Around 95% of overall BAs recirculate back to the liver via portal blood and the remaining percentage is excreted into feces [125].

Moreover, within the gut, a vast number of innate and adaptive immune cells, mainly represented in the colon, are in constant crosstalk with the commensal microbiota and interact with their bacterial metabolites, such as secondary BAs. While the effect of bile acids on the innate immune system has been reviewed elsewhere [128], this review focuses on studies that show how the secondary BAs act as proinflammatory or immunoregulatory factors mediated by adaptive immune cells, Th17 and Treg cells, specifically.

Recent research on BAs has pointed to derivatives of LCA as inhibitors of Th17 cells (3-oxoLCA and isoLCA) and promotors of Treg cells (isoalloLCA) [129,130] (Figure 4). A first in vivo screening of ~30 primary and secondary BAs and their derivatives under Th17- or Treg-cell differentiating conditions pointed to 3-oxoLCA and isoalloLCA as immune regulators [129]. Accordingly, 3-oxoLCA was demonstrated to directly bind to the RORγt ligand-binding domain and inhibit its transcriptional activity. This result confirmed previous studies that demonstrated that the ligand binding domain of RORγt can contain cholesterol or cholesterol-derived molecules, such as hydroxycholesterols, which are highly potent ligands and modulate the differentiation of Th17 cells [131,132]. Subsequent research identified more than 200 bacteria, which reside in the human intestine and belong to the Actinobacteria and Firmicutes phyla, that converted LCA into 3-oxoLCA [130]. In contrast, isoalloLCA increased the expression of *FOXP3* mRNA and decreased the proliferation of effector T cells when Treg cells were pretreated with this BA derivative prior to perform the in vivo suppression assay [129]. Further molecular investigation on their mechanisms of action showed that neither 3-oxoLCA Th17 suppression activity is mediated by vitamin D receptor (VDR) or farnesoid X receptor (FXR), which have been related to bile acid regulation [133,134,135], nor isoalloLCA immunoregulatory activity, which regulates FoxP3 transcription through the CNS3 non-coding enhancer, is mediated via VDR, FXR, or the transcription factor REL [129]. Further experiments elegantly showed how isoalloLCA produces reactive oxygen species (ROS) as by-products of mitochondrial oxidative phosphorylation, which Treg cells rely on for their energy production, and how this mitochondrial ROS production promotes Treg cell differentiation [129]. In vivo experiments demonstrated that both 3-oxoLCA and isoLCA oral administration to wild type mice or mice under gut inflammatory conditions could directly reduce the percentage of ileal Th17 cells [129,130]. Specifically, the 3-oxoLCA effect did not depend on gut microbiota composition or the presence of a specific commensal community [129]. In contrast, isoalloLCA together with 3-oxoLCA, but not isoalloLCA alone, could also directly increase the percentage of Treg cells under these two experimental conditions in a CNS3-dependent manner [129]. Finally, the adoptive transfer of isoalloLCA-treated Treg cells protected mice from developing experimental colitis by decreasing the proliferation of T effector cells to the same extent as those animals that received TGFβ^high^ Treg cells [129].

Other two secondary BAs, isodeoxycholic acid (isoDCA) and ω-muricholic acid (ω-MCA), increased Treg cell frequency when stimulating naïve CD4^+^ T cells in the presence of the professional APCs, DCs [136] (Figure 4). These two compounds, together with nine other secondary BAs, were also tested regarding their ability to modulate proinflammatory Th17 cells; however, none of them were able to do so [136]. Further investigations on isoDCA, the one that is substantially present physiologically, proved that this secondary BA promotes an antiinflammatory state of DCs (lower expression of IFN signaling and antigen processing and presentation genes, among others), being their FXR involved in the induction of Treg cells [136]. Last, in vivo experiments using isoDCA-producing bacteria, which did not alter the overall microbiota metabolism or other immunoregulatory metabolite levels such as SCFAs, showed how these microorganisms had the capability to induce peripheral cTreg cells [136]. This latter finding shows up how microbial metabolism of BAs impacts immune populations in the colon and its potential role in gut-related diseases and treatment.

Other Treg cells with intestinal immunoregulatory capabilities and induced by gut commensal microbiota are RORγ^+^ cTreg cells [137]. Providing rich versus minimal diet to SPF and GF mice showed how the lack of nutrient-rich diet for SPF animals decreased RORγ^+^ cTreg cells while accumulating lower levels of deconjugated primary BAs and secondary BAs compared to SPF-rich diet mice [138]. Moreover, providing a nutrient-rich diet to GF animals resulted in the lowest proportion of this regulatory T cell population when compared to SPF mice and a significant accumulation of conjugated primary BAs while no downstream metabolites were produced due to a lack of gut microbiota [138]. When SPF mice were supplemented with a mixture of certain primary (CA or CDCA) or secondary (UDCA, LCA, or 3-oxoLCA) BAs, a significant and specific increase of RORγ^+^ cTreg cells was observed [138]. However, dietary factors are not the only element that impact BA metabolism and subsequent cTreg cells, but microbial metabolic pathways related to primary BA deconjugation were proven to be essential [138]. The most abundant BA receptor (BAR) in the colon, VDR, whose genetic variants have been associated with IBD [139], was proven to have a major role in the modulation of RORγ^+^ cTreg cells [138]. Finally, since this immunoregulatory cell type has been related to colonic homeostasis and colitis improvement [140], the authors wanted to test the role of BAs on colonic inflammatory responses. The maintenance of RORγ^+^ cTreg cell population before colitis induction was mediated by the BA-VDR axis and proven to be important for alleviating experimental disease signs [138]. Indeed, since secondary BA rates have been described to be decreased in IBD patients and further demonstrated to elicit antiinflammatory responses in vivo [130,141], treatment with these BAs could lead to a decrease in the chronic inflammation of IBD.

## 3. Conclusions and Future Directions

The symbiotic relationship between the gut microbiota and humans shows that not only the presence of these microorganisms but also the metabolites they produce have a fundamental role in the host well-being. The bioavailability of some of these metabolites falls on specific gut microorganisms, so gut dysbiosis has direct and detrimental consequences to host homeostasis. Specifically, some microbiota-derived metabolites modulate adaptive immune populations such as Th17 and Treg cells, thus affecting immune regulation and being involved in immune-related or immune-mediated diseases such as colorectal cancer, MS, or IBD. Indeed, decreased amounts of different metabolites have been described in some of these disorders.

In contrast, the lack of gut microbiota-derived metabolites could be rebalanced by the exogenous administration of these molecules or other compounds that bind to their immunoregulatory receptors in a homologous manner. A workflow proposed by Dorrestein and collaborators, synthesis-based reverse metabolomics, aims to screen the chemical variants of different families of compounds of interest and connect those with diverse biological phenotypes [142]. First, combinatorial synthesis is applied to generate new compounds based on specific compounds of interest that will be modified by a series of simple chemical reactions: additions, oxidations, or reductions, among others. Afterwards, mass spectrometry (LC-MS/MS) spectra are collected and searched against public repositories, so newly generated libraries are analyzed and can be classified by organism, tissue, or disease state, among others. Another alternative to find novel target–ligand combinations involves in silico modeling [143]. Once the 3D structure of a protein of interest predicts the interaction between that target and the potential molecule, a subsequent computational generation and optimization of alternative mimics will predict better binders. In this way, the development of chemical libraries composed of novel microbial-derived metabolites might be an option to target these immune pathways and rebalance altered adaptive immune responses.
